# CRISPR-Cas9: how research on a bacterial RNA-guided mechanism opened new perspectives in biotechnology and biomedicine

**DOI:** 10.15252/emmm.201504847

**Published:** 2015-03-21

**Authors:** Emmanuelle Charpentier

**Affiliations:** 1 Department of Regulation in Infection Biology, Helmholtz Centre for Infection ResearchBraunschweig, Germany; 2 Department of Molecular Biology, The Laboratory for Molecular Infection Medicine Sweden (MIMS), Umeå Centre for Microbial Research (UCMR), Umeå UniversityUmeå, Sweden; 3 Hannover Medical SchoolHannover, Germany

CRISPR-Cas9 emerged as a powerful and universal technology for genome engineering with wide-ranging innovative implications across biology and medicine. The technology is based on RNA-programmable molecular scissors to perform precise surgery on genes. Various versions of the system have been developed to broaden its range of applications to manipulate genes and their expression in a large variety of cells and organisms (reviewed by Doudna & Charpentier, 2014; Hsu *et al*, 2014). CRISPR-Cas9 has quickly been adopted by biologists and is now recognized as a transformative technology in various fields of research with the potential to benefit the understanding and treatment of human genetic disorders, cancers and infectious diseases (reviewed by Doudna & Charpentier, 2014; Hsu *et al*, 2014).

The origin of the CRISPR-Cas9 system, also referred to as the type II CRISPR-Cas system (reviewed by Barrangou & Marraffini, 2014), lies in an ancient immune mechanism in bacteria. Bacteria are subjected to diverse environments and constantly face heavy assaults by bacteriophages or other types of mobile genetic elements (Fig1). CRISPR-Cas is one of the various defense mechanisms that bacteria have evolved to fend off attacks by foreign elements. The CRISPS-Cas system is based on one or two types of RNA components (the CRISPR RNAs or crRNAs for all CRISPR-Cas systems, and a second RNA called tracrRNA in the case of the type II system) and protein components (the CRISPR-associated or Cas proteins) that work together in ribonucleoprotein complexes to interfere with the invading nucleic acids (Fig1). There are two events of interaction with the invaders: memorization of a genetic element upon a first infection (adaptation) and destruction of the same element upon a second infection (interference). In adaptation, the invading mobile element is recognized by Cas proteins that insert a short sequence of the invader into a CRISPR array located on the bacterial genome. The integrated sequences constitute a genetic memory that effectively prevents the host from being re-infected. The library of memorized invader sequences is transcribed into an RNA molecule that undergoes one or two processing events to generate short mature crRNAs, each containing a piece of a memorized genetic element. In interference, the crRNAs are incorporated with Cas protein(s) into CRISPR ribonucleoprotein complexes (crRNPs), guiding the sequence-specific degradation of cognate invading DNA or RNA.

**Figure 1:**
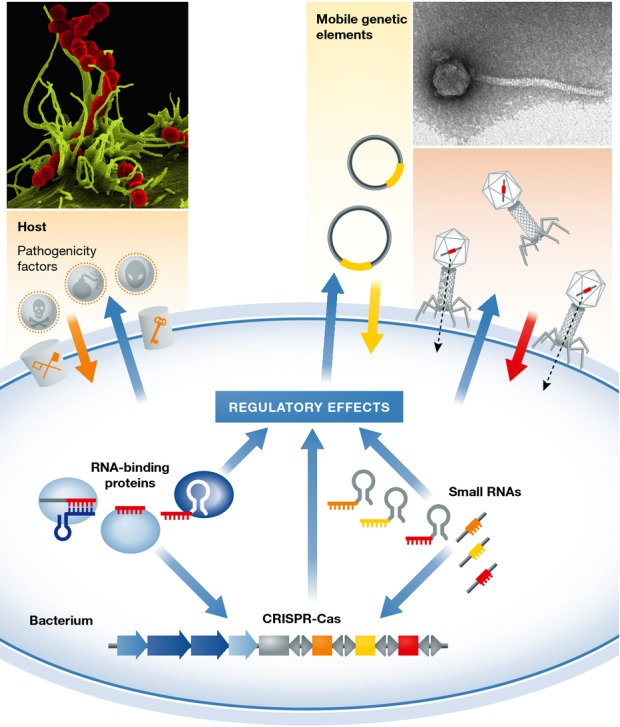
Regulatory functions of CRISPR-Cas systems in bacteria The CRISPR-Cas system is an RNA-mediated adaptive defense system against mobile genetic elements such as phages or plasmids. In the case of *Streptococcus pyogenes*, the type II CRISPR-Cas system (CRISPR-Cas9) influences the virulence potential of the human pathogen by limiting the acquisition of virulence genes carried on temperate phages. It has also been reported that in, for example, *Francisella novicida*, the type II CRISPR-Cas system has evolved a regulatory function in the virulence of the human pathogen, independently of its role as an immune system. Ribonucleoprotein complexes encoded by CRISPR-Cas systems target the DNA or RNA of invading genomes (adaptive immune function against mobile genetic elements, bacteria and archaea) and were also reported to target mRNAs (regulatory function in endogenous gene expression, e.g., *F. novicida*). Electron micrographs, courtesy of Manfred Rohde, HZI, Braunschweig, Germany.

In the arms race between bacteria and their predators, the CRISPR-Cas systems have highly evolved. Current nomenclature classifies CRISPR-Cas into three main types with eleven subtypes. Both type I and type III CRISPR-Cas systems can be found in bacteria and archaea, whereas the type II CRISPR-Cas system has only been identified in bacteria. All CRISPR-Cas systems share the common principle of adaptive immunity as described above. However, the various subtypes with their diversity of Cas proteins operate by using distinct mechanisms to perform each of the three steps outlined above: memorization, crRNA biogenesis and interference. Interference in type I and type III CRISPR-Cas systems involves crRNPs that are each composed of a guide crRNA and a complex of Cas proteins. In contrast, the targeting type II CRISPR-Cas system is specified by a crRNA duplexed with an additional RNA, the *trans*-activating CRISPR RNA (tracrRNA), forming a DNA interfering complex with one single protein, Cas9 (formerly named Csn1). It is the simplicity and RNA programmability of the CRISPR-Cas9 system (one protein guided by a duplex of RNAs) that has enabled its straightforward application to site-specifically target any genomic location of virtually any cell and organism.

The streptococcal type II CRISPR-Cas system (also known as type II-A) was one of the first CRISPR-Cas systems to be studied in the laboratory. The first experimental evidence of an adaptive immune function of the system was demonstrated in type II-A in *Streptococcus thermophilus* (Barrangou *et al*, 2007). Follow-up studies in this bacterial species defined experimentally the critical role of the so-called PAM sequence (protospacer adjacent motif) in the recognition between self and non-self (Deveau *et al*, 2008) and the *in vivo* targeting and cleavage of invading phage and plasmid DNA by the type II-A system (Garneau *et al*, 2010; Sapranauskas *et al*, 2011).

The identification and deciphering of the role of tracrRNA in the human pathogen *Streptococcus pyogenes* is considered a hallmark in the understanding of the mechanism involved in the type II CRISPR-Cas system. This research was initiated by a genome-wide computational analysis aiming to reveal novel small RNAs with regulatory functions in the virulence of this human pathogen. In the CRISPR-Cas immune pathway, tracrRNA is an essential component of the ribonucleoprotein CRISPR-Cas9 complex, acting as a *trans*-activator required for processing crRNAs (Deltcheva *et al*, 2011), and also as an essential component of the complex that targets cognate DNAs (Jinek *et al*, 2012). Remarkably, tracrRNA has also evolved a regulatory function in endogenous gene expression. In the human pathogen *Francisella novicida*, tracrRNA regulates the expression of virulence factors by guiding a complex formed by the duplex tracrRNA:scaRNA and the protein Cas9 to virulence factor mRNAs (Sampson *et al*, 2013). This alternative CRISPR-Cas9 complex could provide a means to site-specifically silence mRNAs in cells and organisms.

The deciphering and harnessing of the CRISPR-Cas9 system is yet another example of how research on bacterial mechanisms has benefited the fields of biotechnology and biomedicine. Bacteria have revolutionized molecular biology as an almost unlimited source of enzymes, and they are extensively used in industry in various ways (for example, for the manufacture of diary products, and the production of biological substances such as enzymes, vaccines, antibiotics and biofuels). Bacteria were the first forms of life to appear on earth and they are essential for our existences at multiple levels. Yet, we are far from understanding the breadth of bacterial diversity and the multiple mechanisms they have evolved to interact with their environment, including extracellular environment, predators and hosts. There is a great need to increase our knowledge of the fundamental molecular mechanisms involved in bacterial physiological processes that include adaptation of bacterial cells to changing environmental conditions, cellular communication, metabolism and pathogenicity. Only a deeper comprehension of how regulatory proteins, RNAs and other molecules operate in the cells will help us unravel new molecular principles for possible translation into novel biotechnological applications such as genome editing tools, and novel biomedical applications such as therapeutic genome editing and anti-infective strategies.

The widespread and quick adoption of the custom-engineered sequence-specific nuclease CRISPR-Cas9 for routine use is to be considered in the context of an increasing need for universal and versatile technologies that enable the introduction of site-specific changes in genomes and the precise manipulation of genes and their expression. Many of the earliest approaches of genome editing relied on the principle of site-specific recognition of DNA sequences, first by oligonucleotides, small molecules or self-splicing introns, followed by site-directed homing endonucleases, zinc finger nucleases (ZFNs) and TAL effector nucleases (TALENs) (reviewed by Damian & Porteus, 2013). However, difficulties of design, synthesis and validation of these nucleases remained an obstacle to widespread applications. After decrypting the DNA targeting mechanism of the CRISPR-Cas9 system in 2012 (Jinek *et al*, 2012), it became quickly evident that the RNA-programmable Cas9 system would offer significant advantages, primarily versatility, efficacy, specificity and ease of use, compared to the previous gene editing systems such as ZFNs and TALENs (Jinek *et al*, 2012). The scientific community immediately applied CRISPR-Cas9 in a plethora of cells and organisms and further developed the technology, offering a versatile toolbox for a broad range of applications (reviewed by Doudna & Charpentier, 2014; Hsu *et al*, 2014). The CRISPR-Cas9 technology, which is being widely adopted by academics and by research and development organizations of the pharmaceutical and biotechnology industry, provides a unique opportunity for the scientific and medical community to translate this gene editing tool into human therapeutics. This includes gene therapy, for example by correcting gene defects in progenitor cells in devastating genetic diseases, and the development of CRISPR-Cas9 as potential treatment for chronic diseases such as cancer and HIV.

## References

[b1] Barrangou R, Fremaux C, Deveau H, Richards M, Boyaval P, Moineau S, Romero DA, Horvath P (2007). CRISPR provides acquired resistance against viruses in prokaryotes. Science.

[b2] Barrangou R, Marraffini LA (2014). CRISPR-Cas systems: prokaryotes upgrade to adaptive immunity. Mol Cell.

[b3] Damian M, Porteus MH (2013). A crisper look at genome editing: RNA-guided genome modification. Mol Ther.

[b4] Deltcheva E, Chylinski K, Sharma CM, Gonzales K, Chao Y, Pirzada ZA, Eckert MR, Vogel J, Charpentier E (2011). CRISPR RNA maturation by trans-encoded small RNA and host factor RNase III. Nature.

[b5] Deveau H, Barrangou R, Garneau JE, Labonté J, Fremaux C, Boyaval P, Romero DA, Horvath P, Moineau S (2008). Phage response to CRISPR-encoded resistance in Streptococcus thermophilus. J Bacteriol.

[b6] Doudna JA, Charpentier E (2014). Genome editing. The new frontier of genome engineering with CRISPR-Cas9. Science.

[b7] Garneau JE, Dupuis MÈ, Villion M, Romero DA, Barrangou R, Boyaval P, Fremaux C, Horvath P, Magadán AH, Moineau S (2010). The CRISPR/Cas bacterial immune system cleaves bacteriophage and plasmid DNA. Nature.

[b8] Hsu PD, Lander ES, Zhang F (2014). Development and applications of CRISPR-Cas9 for genome engineering. Cell.

[b9] Jinek M, Chylinski K, Fonfara I, Hauer M, Doudna JA, Charpentier E (2012). A programmable dual-RNA-guided DNA endonuclease in adaptive bacterial immunity. Science.

[b10] Sampson TR, Saroj SD, Llewellyn AC, Tzeng YL, Weiss DS (2013). A CRISPR/Cas system mediates bacterial innate immune evasion and virulence. Nature.

[b11] Sapranauskas R, Gasiunas G, Fremaux C, Barrangou R, Horvath P, Siksnys V (2011). The *Streptococcus thermophilus* CRISPR/Cas system provides immunity in *Escherichia coli*. Nucleic Acids Res.

